# Smart Homes Supporting the Wellness of One or Two-Person Households

**DOI:** 10.3390/s22207816

**Published:** 2022-10-14

**Authors:** Myung Eun Cho, Mi Jeong Kim

**Affiliations:** 1 Construction Research Institute, Hanyang University, Seoul 04783, Korea; 2 School of Architecture, Hanyang University, 222 Wangsimni-ro, Seongdong-gu, Seoul 04783, Korea

**Keywords:** smart home, wellness, one or two-person household, user experience, survey, scenario

## Abstract

The reduction in face-to-face contact and the increase in time spent at home during the ongoing coronavirus disease pandemic have resulted in increasing interest and demand for smart homes. Further, the rapid increase in the number of one-person and two-person households in Korea recently has led to these becoming representative household types. This study identifies the wellness characteristics of such households and proposes a direction for smart home development to help them lead healthy, happy lives. It focuses on mapping residents’ perceptions and experiences to scenarios and on identifying the functions required in smart homes and the technologies needed to provide these functions. It uses data from a survey to investigate and analyze the wellness characteristics of one- and two-person households in five dimensions and develops five scenarios of representative household types. By analyzing the developed scenarios, this study proposes smart homes that support the wellness of such households in six categories: exercise/sports, hobby/entertainment, social communications, occupation/work, self-development/education, and energy conservation. These households are exposed to digital environments from an early age and are familiar with the internet and technologies. Therefore, they are likely to adopt innovative technologies in housing. Thus, the smart home development proposed in this study is a promising strategic approach to housing planning.

## 1. Introduction

Computing is being embedded in an increasing number of spaces to support daily lives following the advancement of information and communication technologies, the Internet of Things (IoT), artificial intelligence (AI), and big data technology. Owing to the recent development of 5G and virtual technologies, such as augmented reality (AR) and virtual reality (Peek, #27), the metaverse is widely used in society and the economy, and currently, online experiences, such as games and social networking services in the metaverse are connected with various activities in the real world [1]. During the coronavirus disease—or COVID-19—pandemic, measures such as social distancing and a shift to working from home and conducting classes online have been implemented, resulting in people being forced to stay home for long periods. As a result of these changes, the interest in and the importance of smart homes has further increased. Research on smart homes has developed to such an extent that appropriate customized services can be provided by collecting and analyzing information about residents through sensors and monitors by combining IoT, AI, and big data technologies [2,3,4].

Recently, the number of households with three or more members, that is, those consisting of a couple and their unmarried children (31.7% of the total number of households in 2020), has decreased in Korea, and the number of households with one or two persons has been increasing rapidly. In 2020, one-person households accounted for 62.1% of the total number of households in Korea [5]. The increasing trend of one- and two-person households is expected to continue for the next several years, and the research on housing for them should be conducted from a more diverse perspective than that adopted in the literature at present. Distinct from earlier generations, they have been exposed to the digital environment from an early age, are proficient in using the internet, and are accustomed to sharing interests and producing content through social networking services [6]. Along with technological innovations, a smart home is expected to provide them with a home where they can enjoy a happier, healthier life.

To date, research on smart homes has focused on the development and the introduction of advanced technologies and has not based these processes on understanding the users [7]. In addition, despite the advantages of applying technology to create smart homes, many users have not accepted or actively used such technology, resulting in a high rate of rejection of smart devices [8,9]. However, smart homes cannot be created only through the development and application of technology. Research on smart homes should be conducted from a user-centric perspective to ensure that users accept such homes, and the user experience (Wilson, #10) must be considered for developing advanced smart homes. Therefore, the aim of this study is to understand various aspects of UX, including residents’ emotions and cognition, by applying the concept of wellness to the smart home. Then, using this insight, this study suggests the types of smart homes that one- or two-person households need to lead a healthy, happy life.

## 2. Theoretical Considerations

For the theoretical consideration, we first review how the smart home research has been developed, what aspect they have focused on, and what target groups they have dealt with to identify the gap the previous research could not figure out. Then, we include the theory of wellness that we need to pay attention to in the smart home research because smart homes need to support a healthy living environment, including the dimensions of wellness for all ages.

### 2.1. Smart Home Development

According to the initial concept of a smart home, it is an environment that includes technology to control devices and systems automatically, including lighting, air conditioning, and home appliances, and to provide security, such as through access control and alarm systems [10]. Later, as home networking developed after the spread of high-speed internet, sensors were gradually installed in everyday objects and interlocked with mobile devices, thereby expanding the smart home concept [11]. The main components of a smart home are intelligent technologies, such as remote monitoring systems, context awareness, and AI. Through these, communication and information collection between intelligent devices, things, and humans are possible, which allows for the analyses of residents’ life patterns. IoT technology supports access to smart devices and the remote monitoring of embedded devices, and AI applies a visual and sensory-based tracking system to identify users through the recognition of facial expressions and emotions [12,13,14].

Typically, the subjects of smart home research are chronically ill or older adults who find it challenging to live independently. The primary function of the smart home is to monitor their condition and manage the diseases they have, in order to help them lead independent lives [15,16]. By monitoring the cognitive status and the physical condition of the elderly through intelligent devices, it is possible to detect their health problems in advance. Medical services can be provided to them through recognition of changes in their gait and behavioral patterns using technologies such as sensors and algorithms installed in smart homes, which detect falls and allow families and medical personnel to monitor their health remotely [17]. In addition, telemedicine, which connects patients and clinicians and thus enables the latter to monitor the former’s physiological signals, such as their heart rate, through wearable devices attached to clothing or skin or to manage chronic diseases at home, is becoming common [18,19,20]. However, smart homes need to be extended to provide a healthy and intelligent living environment for all ages and not just the elderly or patients. Many researchers have critically reviewed previous research on smart homes [7,11,21]. Some also found that potential users of innovative home services have diversified recently [22] and user perceptions of adopted technologies differ depending on their interest in the quality of life [23].

### 2.2. Wellness

The term wellness focuses on an interrelational and holistic perspective, which represents a shift from the simple meaning of health [24]. Earlier definitions of health viewed the body as an isolated physiological system free from illness and disease [25]. However, in line with the changing needs of the times, the holistic view has completely changed this concept of health. The concept of wellness is essential to a state of well-being free from disease. Wellness refers not only to the absence of negative factors, such as disease, but also to the presence of positive factors, such as physical health and happiness [26,27]. The meaning of health in current society has become more complex and is correlated mainly with well-being, quality of life, life satisfaction, and happiness [28]. The concept of wellness continued to evolve until it was an expanded concept of health, encompassing all aspects of a person (mind, body, spirit) [29]. Coulter (1993) described wellness as a way of life in which the mind, body, and spirit are in harmony [30]. Kirsten et al. (2009) stated that wellness includes various dimensions, such as mind, body, spirit, and community interaction, and that these factors are interconnected [31]. Ardell (2005) emphasized cognitive processes to promote happiness and satisfaction in people’s diverse lifestyles and in various intellectual, physical, emotional, and occupational domains [32].

The concept of wellness allows an individual to be viewed from a holistic point of view and is used in research in various fields as a multidimensional theoretical framework [31]. The Aware Home Research Initiative, Georgia Tech’s interdisciplinary housing research team, conducted a study on the development and application of the technology needed for residents, focusing on the wellness model for happy, healthy living at home [33]. Depending on the researcher, the dimensions of wellness are somewhat different. The intellectual and mental dimensions are sometimes integrated or separated, and the importance of the occupational and environmental dimensions differs depending on the researcher as shown in Table 1. The wellness model generally comprises several dimensions, such as physical, spiritual, emotional, social, intellectual, occupational, and environmental, and ensuring a balanced life between these dimensions is important.

The concept of wellness, which emphasizes the balanced interconnectedness of human beings at different levels, can help people select the factors to consider in the smart home for improving the quality of life in their homes and in their daily lives. Therefore, given that it is important to consider the needs of the growing population of one- or two-person households in Korea, this study uses the concept of wellness in proposing the development of a smart home in which these households can live a happy, healthy life in multiple dimensions.

## 3. Methodology

A survey and a scenario method were used in parallel to conduct a user-centered smart home study. The survey analyzed the wellness characteristics of one- or two-person households in five dimensions, and using the analysis results, a scenario of five representative types was developed.

### 3.1. Questionnaire Survey

One-person and two-person households consisting of retired individuals and the elderly were excluded from the survey. Two surveys were conducted for this study, one for one-person households and the other one for two-person households. Only those who live alone because they are financially independent from their parents’ generation were selected for the survey on one-person households. Given the high possibility of a difference in wellness characteristics according to gender, stratified sampling was performed by gender. This survey was conducted in October 2019 and completed questionnaires from 100 individuals were included in the analysis. Although two-person households may include elderly households, single-parent households, and grandparent households, this study includes two-person households consisting of couples with no children after marriage.

A preliminary survey showed that even in the same household, the responses from the husband and the wife differed. Therefore, only homemakers—all of whom were women—were included in the survey. This survey was conducted in July 2020 and completed questionnaires from 64 women were included in the analysis. The surveys were conducted online using the snowball sampling technique. Snowball is a nonprobability sampling method suitable for research targeting specific households. The subject recruit starts from among their acquaintance, and the sample group grows similarly to a rolling snowball [41]. The SPSSWIN 23 statistical program was used to calculate the frequency and the mean and to conduct tests for reliability and cross-analysis, a *t*-test, and an analysis of variance test. The next section presents the wellness measurement tool developed in this study.

### 3.2. Wellness Measurement Tool

This study used the wellness concept of prior studies to develop a tool for measuring the wellness characteristics of one- and two-person households in Korea. In all, 14 items were derived in five dimensions: Physical, Social & Environmental, Intellectual, Occupational, and Spiritual & Emotional. Using these items, 26 questionnaire items were developed (see Table 2 for details). The internal reliability analysis of the developed items showed that the Cronbach α value was 0.6 or higher (Table 6). The measure of wellness was evaluated on a 5-point Likert scale (1 point: not at all~5 points: very much), and the results were analyzed.

To ensure Physical wellness, individuals need to engage in healthy physical activity through exercise and diet. Social & Environmental wellness relates to a sustainable environment, which emphasizes the interdependence of humans and nature and their contribution to improving community life. It includes factors such as participation in the local communities and environmental conservation activities and the use of community facilities and green spaces. Intellectual wellness relates to expanding and developing one’s skills and knowledge through creative activity, continuous stimulation, and inquiry. It includes one’s level of intellectual curiosity and various cultural activities and the growth through them. For example, people can be interested in recent social issues; pursue personal interests; read books, magazines, and newspapers; or participate in creative activities. Occupational wellness refers to obtaining life satisfaction and abundance through work and includes factors such as satisfaction with one’s work, recognition, and reward from others. A premise of occupational health is that occupational development is related to one’s attitude toward work. Ensuring Spiritual & Emotional wellness is a continuous process of finding the meaning and purpose of one’s life, for which maturity and development are essential. High self-esteem, a positive attitude toward life, and the ability to cope with stress are related to affection for human relationships, family, and society, and the consideration of existence and the meaning of life. On the spiritual level, people act according to their beliefs and values and find joy and happiness in their spiritual life. On the emotional level, it is important to freely express one’s emotions and manage them effectively. Emotional health also includes managing negative emotions and behaviors, such as anxiety, depression, and loneliness. Next, the scenario technique used in this study is described.

### 3.3. Scenario Method

The survey results were used to develop scenarios by identifying five representative types of virtual residents. The scenario method is an overall accepted research method in housing studies [42,43]. A feature of this method is that it can effectively predict future life, which suggests the possibility of determining appropriate technology use and the right direction for users. Problems were derived through the developed scenarios, and customized housing services and solutions were proposed. The scenarios were developed by considering the ways that the services for which technologies are installed and used could be used according to the circumstances and needs of the occupants. The future smart home will be an environment that is intelligent and interacts with humans. To develop a sustainable smart home that allows its residents to lead a happy, healthy life, it is necessary to understand the setting of these residents and provide appropriate information and services. Therefore, this study applied the scenario technique to smart home development. The next section presents the results of this study.

## 4. Results

### 4.1. Survey Results

The survey respondents were from one-person and two-person households, and they were engaged in economic activities. The average age of those from the former and the latter households was 31.42 years and 39.79 years, respectively (Table 3). Individuals in their 20s and 30s comprised about 86% of the one-person household sample, and those in their 30s comprised 68% of the two-person household sample.

Table 4 shows the results on analyzing the housing characteristics of the survey participants. They lived in apartment types rather than in detached houses. In particular, 50% of one-person households lived in tenements and multifamily houses, and 81.2% of two-person households lived in apartments. They were more likely to rent a house than to own one. The ownership rate of one-person households was 6.0%, which was significantly lower than that of two-person households (40.6%). The average size of the house for both household types was 66.72 m^2^, which is not large. In particular, it was found that, on average, those in a one-person household lived in a house of 53.72 m^2^, and those in a two-person household lived in a house of 87.08 m^2^. Further, the two-person households lived in houses with an average area of about 33 m^2^ larger than that of a single-person household. This difference is likely attributable to the fact that many of the one-person households lived in a one-room type unit.

Table 5 shows the results regarding the surveyed households’ interaction with the residents of neighboring houses within their apartments. In all, 77.4% of the respondents did not interact with their neighbors, and more two-person households (95.3%) responded that they did not interact with neighbors than one-person households did (66.0%). However, both household types wanted to feel intimacy, or to belong through interaction, with neighbors and to share useful information with them.

#### 4.1.1. Wellness of One- and Two-Person Households

**Figure 1:**
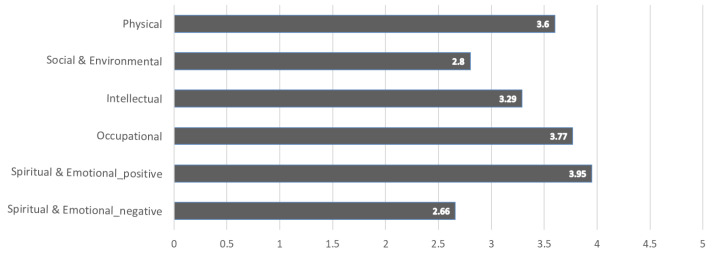
Wellness in the Five Dimensions of One-person and Two-person Households.

An analysis was performed of the wellness of the one-person and two-person households. We first combined two household types of perceptions of wellness to identify the average wellness scores in five dimensions, as shown in Figure 1. Positive and negative questions were both included in the Spiritual & Emotional dimension; the positive questions were about more desirable aspects and had higher scores, whereas the negative questions were about less desirable aspects and had lower scores. The score on the Spiritual & Emotional dimension was the highest, followed by the Occupational dimension with 3.77 points, and the Physical dimension with 3.60 points. Conversely, the average score on the Social & Environmental dimension was the lowest at 2.80.

Table 6 shows the results for each wellness item for both household types. “PH1-1. I am currently exercising regularly” had the highest score with an average of 4.13 points. The Social & Environmental dimension score was lower than that of the other dimensions, but in particular, “SE2-1. I am involved in volunteer work or local community activities” had the lowest score of 1.81. “SE1-2. I get along with my neighbors” had a score of 2.12, and the score for “SE4-1. I use local sport facilities and community service centers” was low, at 2.40. At the Intellectual level, the average score of the item related to acquiring knowledge and skills, “IN3-2. I tend to constantly try to develop myself,” was the highest at 3.73; “IN1-1. I watch movies, performances, and exhibitions” had a score of 3.60 points, and “IN1-2. I enjoy hobbies and cultural activities” had a score of 3.67 points. In particular, “OC2-1. I can be more appreciated by others when I work in my area” was 3.88 points, and “OC1-2. I want to succeed in my job” had the highest score of 3.85. In the Spiritual & Emotional dimension, “SM1-1. The reward and love through human relationships are essential to my life” had a score of 3.94, and “SM1-2. I act according to the values and beliefs of my life” showed a high score of 3.94. This study found that one-person and two-person households were emotionally stable and did not have high negative emotions.

#### 4.1.2. Comparison of Wellness between One-Person and Two-Person Households

**Figure 2:**
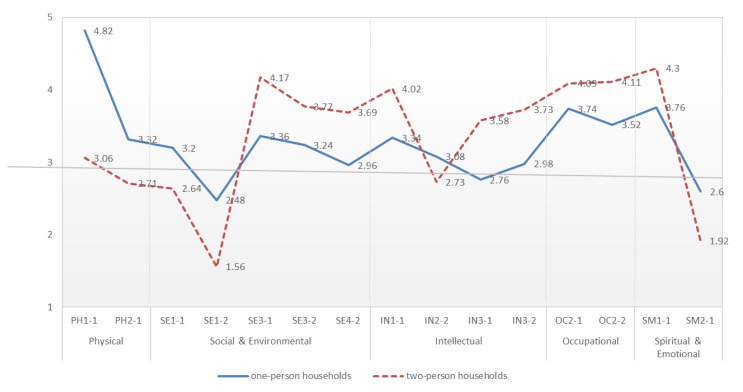
Comparison of Wellness Between One-person and Two-person Households.

The analysis results showed a partially significant difference in wellness between one- and two-person households (Figure 2). An analysis of the Physical dimension revealed that one-person households exercised and ate regularly, distinct from two-person households. In the analysis at the Social & Environmental level, both household types scored low on the SE1 item about interaction with other people. However, the two-person households had lower scores than the one-person households, which indicates that the former found it more difficult to get along with their neighbors than the one-person households did. Conversely, for SE3 items related to the environment, the score of the two-person household was higher than that of the one-person households. For the SE4 item, it was found that the two-person households used surrounding greenery or local community facilities more frequently than the one-person households did.

In the Intellectual dimension, there were significant differences in several items between both household types. Among the IN1 items related to acquiring and stimulating intellectual activity, for “IN1-1. I watch movies, performances, and exhibitions,” the score of the two-person households was higher than that of the one-person households. In addition, the score of the former was higher than that of the latter for IN3 items related to expanding own knowledge and skills. The results imply that the two-person households strived harder than the one-person households did to expand their knowledge and skills. In the Occupational dimension, both household types had high scores. A comparison of both types showed that two-person households had higher recognition and satisfaction with their jobs than one-person households had.

In the Spiritual & Emotional dimension, both one-person and two-person households scored high on items measuring positive aspects but not on questions measuring negative aspects. Certain differences were found in the scores for two items on comparing both household types. For “SM1-1. The reward and love through human relationships are an important part of my life,” the score of the two-person households (4.30) had a higher score that the one-person household (3.76). For “SM1-2. I act according to the values and beliefs of my life,” the one-person households (4.02) had a higher score than the two-person households (3.83). The score for “SM2-1. I feel pessimistic and dark in my life,” which asked about negative emotions, was less than 3 for both household types, and the latter households had more negative emotions than the two-person households.

To develop the one-person and two-person household scenarios, an analysis was conducted to determine potential differences in wellness according to the participants’ demographic characteristics, such as age and gender. A partially significant difference was found for certain items (Table 7).

The analysis of the wellness of one-person households by age revealed differences in the Intellectual, the Occupational, and the Spiritual & Emotional dimensions. In the Intellectual dimension, people in their 40s had higher scores than other age groups, whereas people in their 20s and 30s had higher scores for the items related to occupational activities. This result suggests that people in their 40s are less satisfied than the other age groups with their work and occupation as well as with the rewards and the joys of work. In the Spiritual & Emotional dimension, for “SM1-2. I act according to the values and beliefs of my life,” those in their 40s had high scores (4.57 points). An analysis of wellness according to the gender of one-person households showed that women had higher scores than men, especially for the SE3 items related to the environment and resource conservation in the Social & Environmental dimension. In the Intellectual dimension, women had higher scores than the men did for all items except “IN1-2. I enjoy hobbies and cultural activities,” whereas, in the Occupational dimension, men had higher scores than women. In the Spiritual & Emotional dimension, men were more positive than women in interpersonal relationships and had less frustration and stress.

Next, the analysis of the wellness of two-person households by age showed that in the Physical dimension, those in their 40s (3.30 points) and 50s (4.00 points) exercised more regularly than those in their 30s (2.79 points). Regarding the Social & Environmental dimension, the most significant difference was for “SE2-2. I attend clubs or gatherings.” The score of “SE1-2. I get along with my neighbors” was lower for all age groups, and in particular, for people in their 30s (1.36 points) than for the other age groups. People in their 50s showed higher interest than the other age groups in recycling, waste separation, and energy conservation, as revealed by the score of 5 for SE3-2. In the Intellectual dimension, people in their 50s scored the highest on IN3 items asking about efforts to expand their knowledge and skills. This result shows that those in their 50s tended to learn something new or constantly strived for self-development. In the Spiritual & Emotional dimension, there was a significant difference only for negative questions. Those in their 30s felt the least loneliness, whereas those in their 50s felt the most. Those in their 30s had the lowest score (2.52 points) for SM3-1, indicating that they felt less stressed, and the lowest score (2.29 points) for SM3-2, suggesting that they felt less helpless in daily life.

### 4.2. User-Experience-Oriented One- and Two-Person Household Scenarios

**Figure 3:**
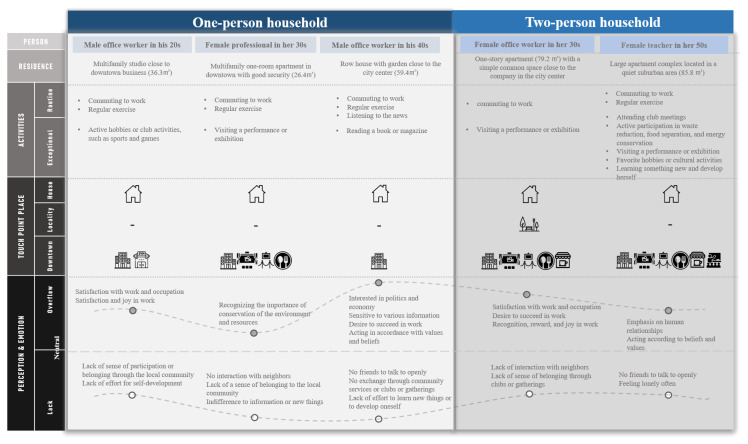
User Experience Mapping Between One-person and Two-person Households.

The results of the questionnaire analysis were used to develop three scenarios for one-person households and two scenarios for two-person households. The scenarios were developed according to the items that differed in the wellness characteristics of both household types, depending on gender and age. The residents’ experience was mapped through context parameters using the activities occurring in the local community centered on the place of residence, the place to visit, and the perceptions and emotions individuals feel (Figure 3). In the experience maps of five persons, the relationships were identified by mapping the places where residents interact in their homes and communities within their own lives. Mapping allows us to analyze the user experience of one or two-person households to find ways to design things that meet their expectations and needs. According to age, gender, and occupation, one-person households were set up with male office workers in their 20s, female professional workers in their 30s, and male office workers in their 40s. Two-person households were developed with female office workers in their 30s and female teachers in their 50s. Scenarios have been developed in which they live in different dwellings and different housing types. For example, a male office worker in his 20s lived in a multi-family one-room apartment of 36.3 m^2^ near the downtown office. The experience map consists of the experiences of residents around three major components. First, the activities experienced by residents were classified into routine daily life and special activities, and the types of activities that would occur were mainly predicted. Second, as a touch point location in a residential area, by area, it was mapped by focusing on the places where residents interact in house, locality, and downtown. Finally, it concerns the perceptions and emotions of residents; this contributed to insights into what customers think and feel when proceeding along the timeline.

Current problems were identified through the context scenarios developed (Table 8). Both household types did not need a large house because they did not have children. They lived in the city center close to their workplace and had convenient transportation options; they led a monotonous life between the company and the house. One problem was that they lacked a suitable space to exercise since they were in a residential area located in the city center. This study considers that were they to have a convenient service for regular meals, they would be able to eat better. In the case of one-person households, they enjoyed playing their favorite soccer games or games with friends, or they enjoyed performances or cultural shows. They wanted to invite their friends to their house, but the space in the house was insufficient for this purpose.

Another problem was that they did not know the residents in the neighborhood. They wanted to interact with their neighbors but found it challenging to do so in their current neighborhood. Although there were community centers, such as parks, gyms, and libraries near their residences, they used few community services and had not participated in many community activities, clubs, or gatherings. They were satisfied with their occupation. They worked hard, and their peers recognized them. In particular, occupational groups, such as professionals and teachers, are increasingly working from home owing to the nature of their work and the ongoing pandemic situation, but their current homes do not have facilities for them to work comfortably. They desire to succeed at work as they age and are constantly striving for self-development; thus, this study considers that the function of housing for education and learning is also necessary.

Women had strong thoughts about the conservation of resources and the environment. They attempted to reduce waste and save energy. Those in their 40s and 50s who were tired of work wanted to do something that allowed them to discover something new about themselves. In particular, in the two-person households, women in their 50s wanted to live in a residential area with adequate greenery within walking distance because retirement was near. They often lost contact with their friends as they moved closer to their workplaces and therefore had no close friends with whom they could communicate openly. They considered themselves lonely compared with other age groups. They may have more free time than the other age groups, and hence, for them, planning a life closer to the local residents is desirable.

## 5. Proposal of a Smart Home and a Smart Community

### 5.1. Smart Home for the Wellness of One- and Two-Person Households

From the analysis of the developed scenarios, smart homes that support the wellness of one-person and two-person households are proposed in six categories: exercise/sports, hobby/entertainment, social communications, occupation/work, self-development/education, and energy conservation, as shown in Table 9. The proposed smart home emphasizes the potential of technologies such as AR, VR, AI, and IoT as an interface that supports UX.

Exercise/sports is an essential function of a smart home for one- and two-person households and can be provided through physical and virtual spaces. Smart exercise equipment and programs can support residents’ exercise in a small dwelling, and community facilities. The recent development of technology has enabled the implementation of realistic services through VR and AR. For example, a virtual sports environment can be implemented through a head-mounted display or smart glass, even in a small physical space. People can experience 3D images by wearing a HoloLens or watching the game on a screen covering the entire wall. Posture coaching such as that used in golf and baseball can be provided through a wearable device or camera. By detecting motion through AR, the video system can be controlled according to the users’ exercise ability to give them the feeling of playing a real game.

One- and two-person households enjoyed watching movies and performances, playing sports and games, reading, and listening to music as hobbies. Intelligent devices can support these activities and activate the gathering of residents through community facilities. Through an AR education program, various cultural classes such as plant raising, baking, and cooking, and hobby clubs can be operated. By providing a multipurpose room for these activities, hobbies and cultural activities can be encouraged through community formation among neighbors. Information about clubs and gatherings for fun activities can be provided through the resident platform, and information about programs and reservation schedules can be provided by installing QR codes in each community center.

Most one- and two-person households lived in small complexes with insufficient community facilities. They had no appropriate space to invite friends or to engage with neighbors. They did not interact with their neighbors and did not often use sports spaces or libraries, parks, and green spaces in the local community. Space planning, services, and programs to activate space use are required to form a desirable social network. Smart homes can support social communication. A shared kitchen where residents can cook together, a lounge where residents can socialize while reading or resting, and a multipurpose space for parties and small groups are required as community facilities. Since they are a generation familiar with exchanges in virtual communities, it is possible for them to form a community online. Community revitalization can also promote activities in public facilities. Through the platform, activities in community facilities can be promoted by sharing information about the location, content, and programs of public places, as well as the neighbors who frequent them. Not only should the design of the spaces be considered, but also how these places can be used in the virtual world.

Recently, as non-face-to-face life continues, companies are actively using telecommuting, flexible working systems, and base offices. A metaverse space where a more interactive environment can be provided through an avatar has begun to be used for non-face-to-face interactions. On metaverse platforms, users can collaborate by wearing a VR device or participating through a PC or mobile device. It is necessary to plan a space focused on individuals that secures their privacy and increases work efficiency by utilizing these advanced technologies as various types of smart offices in housing units and community facilities. In the local community, the box-type shared office Telecube, a single-person office equipped with various business facilities, including a reservation system, power supply, and internet network, can be installed in multiple places, such as subway stations and vacant lots. In the case of public smart offices, facilities such as autonomous seating systems, video conferencing, and intelligent lockers can be provided.

A smart home can provide a space for residents’ self-development and education. Technology-enriched environments, distributed learning techniques, high-end video and 3D technologies can enhance learning effects in these spaces. In addition to study spaces in community centers, it is possible to activate small groups among the people who use them and offer self-development services such as study meetings and programs. A virtual learning center for residents can be created, so that they can listen to language and computer lectures from instructors belonging to prominent academies, and an AR education program can be introduced to support education. Book cafés and libraries can provide e-books that residents can read using a tablet or cell phone, as well as magic books combined with an AR system.

The core purpose of a smart home is to provide an automated and intelligent service by connecting home devices to a network based on IoT. Energy can be saved by using smart home appliances, smart plugs, and smart thermostats. Intelligent appliances can provide information on energy use remotely and save energy through situation-specific notification functions, such as device failures and customized functions. If a specific function of a refrigerator that consumes a lot of energy (such as ice making) is set to operate outside of peak hours, an energy cost-saving effect of 2% to 4% will be generated. Lighting can be controlled by remote control or automation, the illumination and lighting time can be adjusted, and energy can be saved by controlling the amount of heat energy and sunlight entering the room with intelligent blinds. Smart plugs can reduce energy by remote monitoring of home appliances and blocking unused standby power. A smart thermostat can induce energy savings by optimizing HVAC (heating, ventilation, and air conditioning) control by analyzing resident behavior patterns and weather conditions.

### 5.2. Smart Community Based on Residential and Neighborhood Environment

**Figure 4:**
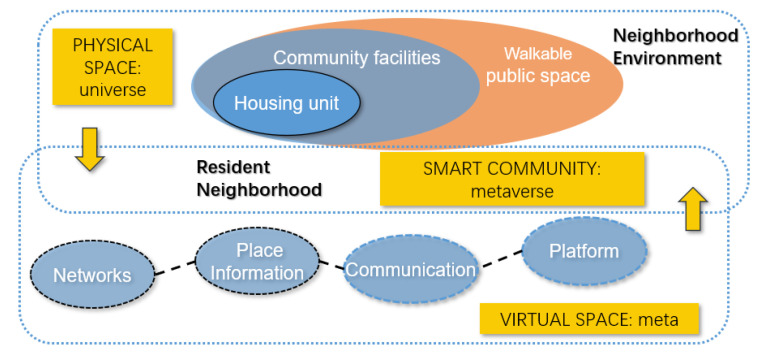
Smart Community Based on Residential and Neighborhood Environment.

Smart homes for one- and two-person households emphasize the social realm among the many functions of a dwelling. Smart homes can improve socialization and help residents overcome their sense of isolation. This can be achieved by designing spaces that support socialization functions and implementing technologies and services. The enabling power of innovative home technology can promote social interaction through face-to-face and non-face-to-face communication. Exercise/sports, hobbies/entertainment, and social communications in the smart home are mainly related to these social domains. This study proposes a smart community model based on residential and neighborhood environments for one- and two-person households (Figure 4).

One- and two-person households live in small areas and form small complexes, so it is difficult to equip them with community facilities. Therefore, the involvement of the public sector is crucial because it can furnish certain community facility functions in public places beyond the minimum facilities available within the complex. The implementation of the concept of the extended housing unit is needed for one- and two-person households because it can include facilities such as parks, gyms, movie theaters, art galleries, and gathering places within a walkable distance from the residence. Extended housing shared by several households in an area can provide a place for various activities to occur among neighbors and where residents can gather.

The shift to the media and virtual societies has also influenced how people create communities and communicate. Young people today find their excitement in placeless communities. Historically, neighborhoods were places where residents’ socialization and sense of community were high. However, localized interactions with neighbors or a geographical community are no longer a prerequisite for building a sense of community. The emergence of virtual communities has also brought negative consequences, such as the cutting off of communication with neighbors in physical spaces and a lack of use of community facilities in the local community. Since proximity and locality can still benefit residents and neighbors, the smart community proposed in this study is distinguished from the general virtual community in that it is a smart community based on proximity and locality. Residents can share a variety of information about neighbors, complexes, community facilities, and public facilities through the platform and can engage in various activities in community facilities with neighbors they meet through the platform.

Therefore, the smart community can play a key role in the environment. Good public spaces within areas can provide conduits for communication and social action, and virtual local communities offer opportunities to promote sociability and develop neighborhoods. Residents can naturally promote each other’s opinions and friendships through shared interests in the area, increasing chances for meeting and promoting stationary activities. In smart communities, a sense of community based on locality can increase as public spaces within the community develop and interactions in public spaces are activated. Boundaries between different complexes are not essential, and the residents of local communities will use the community facilities and public facilities in the complex together. The residents will feel that they belong to the community. The next section presents the conclusion to the study.

## 6. Conclusions

The aim of this study was to develop a smart home for the wellness of one- and two-person households. During the recent COVID-19 pandemic, face-to-face contact has decreased oppositely to time spent at home, which resulted in rising interest and demand for smart homes. To face these changes, a strategic approach considering information and communication technologies and more innovative thinking is needed in housing planning. To this end, this study investigated what residents need for a healthy and happy life and suggested a desirable smart home direction.

In this study, one- and two-person households, which are rapidly increasing as representative households in Korea, were investigated to ascertain their living conditions and living experiences and, from this, what kind of smart home planning is needed. The lifestyle and health conditions of one- and two-person households inevitably differ from those of the elderly: thus, their needs for spaces, technologies, and services vary greatly. As a result of analyzing the wellness of one- and two-person households, it was found that they were lacking a balanced life in various dimensions. The score on the physical dimension was the highest, and the score on the social dimension was the lowest; thus, social and intellectual support were needed. This study proposed a smart home direction to support the wellness of one- and two-person households in six categories: exercise/sports, hobby/entertainment, social communications, occupation/work, self-development/education, and energy conservation.

The study developed scenarios in the context of multidimensional wellness based on a survey to establish a user-centered smart home. The proposed scenarios were used to identify how representative persons behave, visit places, and feel within a community, centered on where they live, and then provide customized solutions. The focus was on mapping residents’ perceptions and experiences and identifying the needed functions and technologies of smart homes. Through the scenario analysis, this study suggested appropriate community facilities, public facilities, programs, and services within the community. Solutions should be expanded through more empirical research to realize a smart home for one- and two-person households in the future. In addition, the side effects of smart communities in terms of social relationships and quality of life should be analyzed from a cognitive perspective. In this study, one-person and two-person households were compared in scenario development. There is a limitation in not being able to compare the two groups concerning gender because participants of one-person households are females and males, and participants of the two-person households were only female in the questionnaire survey. Successful smart home development is not only about designing a space but also about creating a meaningful experience within the space—creating an interaction between the space and community members. Technology changes not only how we interact with others but also how we relate to the environment around us. In this sense, the proposed smart community has a strong potential to revitalize local communities by encouraging interactions with technologies and residents’ behaviors. Smart communities are created when people give meaning to the places in which they live through associations with the virtual world. Powerful networking and mobile technologies provide residents with specific opportunities for meaningful community participation, creating sustainable, healthy, and happy community systems. The authors of this study believe that the concept of housing extended to houses, complexes, neighborhood environments, and public facilities in the local community will positively support the community-based smart community. This study identified the primary function and service categories of the smart community, and each service includes potential programs to support community activities.

## Figures and Tables

**Table 1:** Wellness dimension.

Categories	[34]	[35]	[36]	[37]	[38]	[39]	[40]
Physical	✓	✓	✓	✓	✓	✓	✓
Social/Environmental	✓	✓	✓	✓	✓	✓	✓
Intellectual	✓			✓		✓	
Occupational	✓	✓		✓			
Spiritual/Emotional	✓	✓		✓	✓		✓

**Table 2:** Wellness measurement tool.

Category	Contents	
Physical	PH1	Engaging in regular physical activity
	PH2	Maintaining healthy eating habits
Social & Environmental	SE1	Interacting with others and the community
	SE2	Being involved in the community
	SE3	Maintaining a balance between nature and resources
	SE4	Using community facilities
Intellectual	IN1	Acquiring and stimulating intellectual activity
	IN2	Being curious and seeking information
	IN3	Expanding individual knowledge and skills
Occupational	OC1	Gaining satisfaction and enrichment through work
	OC2	Receiving recognition and rewards for work
Spiritual & Emotional	SM1	Being aware of life purpose and values
	SM2	Feeling positive/pessimistic about oneself and life
	SM3	Having the ability to cope effectively with stress

**Table 3:** Age of the Participants.

Categories		Total F(%)	One-Person Households F(%)	Two-Person Households F(%)	Value
Age	20s	44 (26.8)	44 (44.0)	0 (0.0)	*x*^2^ = 49.18 ***
	30s	86 (52.4)	42 (42.0)	44 (68.8)	
	40s	24 (14.6)	14 (14.0)	10 (15.6)	
	50s	10 (6.1)	0 (0.0)	10 (15.6)	
	Total	164 (100)	100 (100)	64 (100)	

*** *p*-value < 0.001.

**Table 4:** House Characteristics.

Categories		Total F(%)	One-Person Households F(%)	Two-Person Households F(%)	Value
Type	Apartment	80 (48.8)	28 (28.0)	52 (81.2)	*x*^2^ = 46.84 ***
	Detached house	4 (2.4)	4 (4.0)	0 (0.0)	
	Multifamily house	62 (37.8)	50 (50.0)	12 (18.8)	
	Studios and flats with shops	18 (11.0)	18 (18.0)	0 (0.0)	
	Total	164 (100)	100 (100)	64 (100)	
Ownership	Own	32 (19.5)	6 (6.0)	26 (40.6)	*x*^2^ = 39.18 ***
	Charter	83 (50.6)	56 (56.0)	27 (42.2)	
	Half charter	17 (10.4)	14 (14.0)	3 (4.7)	
	Monthly rent	29 (17.7)	24 (24.0)	5 (7.8)	
	Others	3 (1.8)	0 (0.0)	3 (4.7)	
	Total	164	100 (100)	64 (100)	
House size (m^2^)		20.22	16.28	26.39	t = −7.78 ***

*** *p*-Value < 0.001.

**Table 5:** Social Exchanges with Neighbors.

Categories		Total F(%)	One-Person Households F(%)	Two-Person Households F(%)	*x* ^2^
Do you have neighbors to exchange in the complex	Yes	37 (22.6)	34 (34.0)	3 (4.7)	19.19 ***
	No	127 (77.4)	66 (66.0)	61 (95.3)	
	Total	164 (100)	100 (100)	64 (100)	
What do you want to achieve through exchanges with neighbors?	Want to feel intimacy or belonging	65 (39.6)	44 (44.0)	21 (32.8)	7.47 *
	Want to share culture, leisure, sports, hobby activities, etc.	33 (20.1)	24 (24.0)	9 (14.1)	
	Want to receive and share useful information	66 (40.2)	32 (32.0)	34 (53.1)	
	Total	164 (100)	100 (100)	64 (100)	

* *p*-Value < 0.05, *** *p*-value < 0.001.

**Table 6:** Wellness of One-person and Two-person Households.

Categories		Total	One-Person Households	Two-Person Households	T-Value	Cronbach α
		Mean				
Physical	PH1-1. I exercise regularly currently	4.13	4.82	3.06	12.52 ***	0.69
	PH2-1. I keep regular mealtimes	3.08	3.32	2.71	4.05 ***	
Social & Environmental	SE1-1. I invite people to play or eat together at home	2.98	3.20	2.64	3.80 ***	0.77
	SE1-2. I get along with my neighbors	2.12	2.48	1.56	5.58 ***	
	SE2-1. I am involved in volunteer work or local community activities	1.81	1.90	1.69	1.11	
	SE2-2. I attend clubs or gatherings	2.79	2.64	3.05	−2.37 *	
	SE3-1. I think it is important to preserve the environment	3.67	3.36	4.17	−4.78 ***	
	SE3-2. I participate in recycling, waste reduction, and energy conservation	3.44	3.24	3.77	−2.81 **	
	SE4-1. I use local sport facilities and community service centers	2.40	2.50	2.27	1.29	
	SE4-2. I use green spaces for walks or relaxation in the community	3.24	2.96	3.69	−3.47 **	
Intellectual	IN1-1. I watch movies, performances, and exhibitions	3.60	3.34	4.02	−4.87 ***	0.75
	IN1-2. I enjoy hobbies and cultural activities	3.67	3.68	3.66	0.17	
	IN2-1. I spend my time reading books, magazines, and news	3.25	3.34	3.13	1.25	
	IN2-2. I am good at collecting various types of information and know a lot	2.94	3.08	2.73	2.46 *	
	IN3-1. I have recently learned something new	3.07	2.76	3.58	−4.79 ***	
	IN3-2. I tend to constantly try to develop myself	3.73	2.98	3.73	−5.33 ***	
Occupational	OC1-1. I am happy with my work and workplace	3.62	3.57	3.70	-0.98	0.78
	OC1-2. I want to succeed in my job	3.85	3.87	3.84	0.17	
	OC2-1. I can be more appreciated by others when I work in my area	3.88	3.74	4.09	−3.07 **	
	OC2-2. Working gives me the reward and vitality of life	3.76	3.52	4.11	−4.49 ***	
Spiritual & Emotional	SM1-1. The reward and love through human relationships are essential to my life	3.96	3.76	4.30	−3.37 **	0.70
	SM1-2. I act according to the values and beliefs of my life	3.94	4.02	3.83	1.27	
	SM2-1. I feel pessimistic and dark in my life	2.33	2.60	1.92	4.25 ***	
	SM2-2. Sometimes I feel lonely	2.73	2.80	2.63	1.02	
	SM3-1. I have become more frustrated or stressed out	2.93	3.04	2.78	1.39	
	SM3-2. I don’t want to do anything because I’m tired and hard	2.65	2.70	2.59	0.50	

* *p*-value < 0.05, ** *p*-value < 0.01, *** *p*-Value < 0.001.

**Table 7:** Wellness of One-person and Two-person Households According to Demographic Characteristics (only statistically significant items are displayed).

Categories		One-Person Households							Two-Person Households			
		Age				Gender			Age			
		20s	30s	40s	F	Male	Female	t-Value	30s	40s	50s	F
Physical	PH1-1	-	-	-	-	-	-	-	2.79	3.30	4.00	4.26 *
	PH2-1	-	-	-	-	3.12	3.52	−2.19 *	-	-	-	-
Social & Environmental	SE1-1	-	-	-	-	-	-	-	2.70	3.30	2.00	3.32 *
	SE1-2	-	-	-	-	-	-	-	1.36	2.00	2.00	7.02 **
	SE2-1	-	-	-	-	-	-	-	1.56	1.30	2.60	4.73 *
	SE2-2	-	-	-	-	2.40	2.88	−2.31 *	2.68	3.30	4.40	15.12 ***
	SE3-1	-	-	-	-	2.88	3.84	−5.18 ***	-	-	-	-
	SE3-2	-	-	-	-	2.76	3.72	−4.67 ***	3.61	3.10	5.00	8.51 **
	SE4-1	-	-	-	-	-	-	-	2.31	2.70	1.60	3.50 *
	SE4-2	-	-	-	-	2.72	3.20	−2.02 *	-	-	-	-
Intellectual	IN1-1	3.04	3.61	3.42	3.82 *	2.76	3.92	−7.13 ***	-	-	-	-
	IN1-2	-	-	-	-	4.08	3.28	4.37 ***	-	-	-	-
	IN2-1	2.95	3.52	4.00	8.07 **	2.80	3.88	−6.42 ***	3.43	3.10	1.80	10.85 ***
	IN2-2	3.00	2.85	4.00	14.03 ***	-	-	-	2.88	1.80	3.00	6.37 **
	IN3-1	-	-	-	-	-	-	-	3.43	3.40	4.40	7.27 **
	IN3-2	-	-	-	-	2.56	3.40	−4.52 ***	3.75	3.10	4.30	12.79 ***
Occupational	OC1-1	3.78	3.50	3.14	3.28 *	4.00	3.13	5.63 ***	-	-	-	-
	OC1-2	-	-	-	-	-	-	-	-	-	-	-
	OC2-1	-	-	-	-	4.00	3.47	3.77 ***	-	-	-	-
	OC2-2	3.57	3.65	3.00	3.27 *	-	-	-	-	-	-	-
Spiritual & Emotional	SM1-1	-	-	-	-	4.20	3.32	4.56 ***	-	-	-	-
	SM1-2	4.04	3.80	4.57	3.93 *	-	-	-	-	-	-	-
	SM2-1	-	-	-	-	-	-	-	-	-	-	-
	SM2-2	-	-	-	-	-	-	-	2.20	3.30	3.80	11.25 ***
	SM3-1	-	-	-	-	2.60	3.48	−4.52 ***	2.52	3.50	3.20	3.16 *
	SM3-2				-			-	2.29	3.40	3.10	5.04 **

* *p*-Value < 0.05, ** *p*-Value < 0.01, *** *p*-Value < 0.001.

**Table 8:** Scenarios for One-person and Two-person Households.

Type of Residents	First Scenario: One-Person Household		Second Scenario: Two-Person Household	
Common issues	Lead a monotonous life between work and home Lack adequate space to exercise in city dwellings Lack space to invite friends Desire to live with neighbors, but do not know the residents in the neighborhood. Do not often use a community center or community service. Do not attend community activities, clubs, or gatherings Need to work from home			
Key concerns	20s male	Exercise Company and work Hobbies or activities, such as soccer, sports, or games	30s woman	Community facilities within the complex Hobbies and cultural activities for couples
	30s woman	Exercise Company and work Cultural performances, such as films, shows, and exhibitions Self-development for success Conservation of resources and the environment	50s woman	Exercise for health Green space for a walk Friends to speak with openly Free time to learn new things Interested in energy saving and environmental conservation Experience loneliness
	40s male	Tired of company work Discover what sparks his passion Perceived relationships with others while living alone		
Perceived value	Exercise, fitness, hobby, sport Community, gathering place Occupation, work, remote Self-development, education Entertainment, friendliness, shared information Social communication, connected people			

**Table 9:** Smart Home for The Wellness of One- and Two-person Households.

Function of Physical and Virtual Space	Space			Technology		
	Housing Unit	Community Facilities	Public Spaces: Walkable, Gathering Places	Services	Programs	Information
Exercise/Sports	Home training Smart fitness device	Fitness GX Room Screen golf Rock climbing	Swimming pool Stadium Playground Indoor gym Concert hall Showroom Park Jogging/walking roads	Support/assist /consultant service Personalized exercise service	Virtual workout program Various exercise contents	Selective sharing or access Information on place, contents, program, and neighborhood Information exchange with neighbors and platforms
Hobbies/ Entertainment	Home entertainment Smart TV Game device	Media room Game room Pet zone		OTT Media service Game contents service	Game platforms	
Social communications	-	Home party room Lounge Book café Shared kitchen Guest room		Resident communication service Community information services	Entertainment/virtual interaction program	
Occupation/ Work	Home Office/ Home School Personal device IP media device	Meeting room Office room	Telecube	Remote access/connect/interact service Home security service	Platforms for participatory decisions Data security program	Protection Security Reliability privacy Data shared to the cloud in real time
Self-development/ Education		Study room Study hall	Library	Consultant/ database/ contents provision service E-book service	Language, computer programming	
Energy conservation	Intelligent environment Smart device/sensor Monitoring robots Heat/gas/electricity/light	-	-	Control/monitor/management service Cost savings/ Bundling and integration of services	Energy consumption and management program Automation IoT platform Smart controller application	

## Data Availability

The data presented in this study are available on request from the corresponding author. The data are not publicly available due to the policy of research projects.
